# Gratings for synchrotron and FEL beamlines: a project for the manufacture of ultra-precise gratings at Helmholtz Zentrum Berlin

**DOI:** 10.1107/S1600577517015600

**Published:** 2018-01-01

**Authors:** F. Siewert, B. Löchel, J. Buchheim, F. Eggenstein, A. Firsov, G. Gwalt, O. Kutz, St. Lemke, B. Nelles, I. Rudolph, F. Schäfers, T. Seliger, F. Senf, A. Sokolov, Ch. Waberski, J. Wolf, T. Zeschke, I. Zizak, R. Follath, T. Arnold, F. Frost, F. Pietag, A. Erko

**Affiliations:** a Helmholtz Zentrum Berlin für Materialien und Energie, Albert-Einstein-Strasse 15, 12489 Berlin, Germany; b DIOS GmbH, Bad Münstereifel, Schmittstraße 41, 53902 Bad Münstereifel, Germany; c Paul Scherrer Institut, 5232 Villingen, Switzerland; d IOM – Leibniz Institut für Oberflächenmodifizierung eV, Permoserstrasse 15, 04318 Leipzig, Germany

**Keywords:** gratings, soft X-ray optics, diffractive optics, metrology

## Abstract

Blazed gratings are of dedicated interest for the monochromatization of synchrotron radiation when a high photon flux is required, such as, for example, in resonant inelastic X-ray scattering experiments or when the use of laminar gratings is excluded due to too high flux densities and expected damage, for example at free-electron laser beamlines. Their availability became a bottleneck since the decommissioning of the grating manufacture facility at Carl Zeiss in Oberkochen. To resolve this situation a new technological laboratory was established at the Helmholtz Zentrum Berlin, including instrumentation from Carl Zeiss. Besides the upgraded ZEISS equipment, an advanced grating production line has been developed, including a new ultra-precise ruling machine, ion etching technology as well as laser interference lithography. While the old ZEISS ruling machine GTM-6 allows ruling for a grating length up to 170 mm, the new GTM-24 will have the capacity for 600 mm (24 inch) gratings with groove densities between 50 lines mm^−1^ and 1200 lines mm^−1^. A new ion etching machine with a scanning radiofrequency excited ion beam (HF) source allows gratings to be etched into substrates of up to 500 mm length. For a final at-wavelength characterization, a new reflectometer at a new Optics beamline at the BESSY-II storage ring is under operation. This paper reports on the status of the grating fabrication, the measured quality of fabricated items by *ex situ* and *in situ* metrology, and future development goals.

## Abstract 2

Establishing a facility for the production of gratings for synchrotron and free-electron laser applications is reported.

## 1. Introduction   

Diffraction gratings are key components in the application of UV, VUV and soft X-ray radiation at synchrotron radiation and free-electron laser (FEL) facilities. In particular, blazed gratings are attractive since their diffraction efficiency is enhanced by up to a factor of two compared with other grating profiles. The availability of blazed gratings became a problem when Carl Zeiss Optronics GmbH decided to resign their fabrication in 2008. Based on a cooperation agreement between Zeiss and the Helmholtz Zentrum Berlin (HZB), a technology line was established in Berlin to provide and develop diffraction gratings for synchrotron, FEL and astronomy applications.

A diffraction grating consists of an ultra-precise super-polished plane or concave substrate with a large number of equidistant grooves ruled on its surface. A special case is the variable-line-spacing (VLS) grating where a dedicated groove density variation in one or two dimensions is defined by a higher-order polynomial (Harada *et al.*, 1984 ▸; Reininger, 2011 ▸). The manufacture of such optical components which have a laminar or a blazed groove profile requires a complex nano-technological process line including ultra-precise metrology, a groove-shaping process of utmost precision as well as ion beam etching (IBE) to etch the final groove profile into the substrate (Nelles *et al.*, 2001 ▸). Such gratings are usually processed on substrates of quartz glass or single-crystal silicon. Gratings for synchrotron radiation applications usually have to withstand a high heat load from undulator sources. Thus, single-crystal silicon is the material of choice for such gratings because quartz glass does not allow sufficient heat transfer for cooling. With this limitation of silicon as substrate, the holographic recording of blazed gratings by the interference of two laser beams (one from the top side and one from the transparent back side of the substrate) as recently described (Steiner *et al.*, 2013 ▸) is impossible.

The state-of-the-art residual slope error of grating blanks is 100–200 nrad r.m.s. (Siewert, 2013 ▸) and their mid-spatial and high-spatial figure error is <0.2 nm r.m.s. Upcoming gratings at the European XFEL require 500 mm lengths with a residual slope error of 50 nrad r.m.s. (Vannoni *et al.*, 2013 ▸).

Thus such substrates need to be inspected by dedicated metrology instrumentation before manufacturing as well as between each process step. While the holographic ruling of laminar gratings is a well known process (Schmahl & Rudolph, 1970 ▸, 1974 ▸; Hutley, 1976 ▸; Lemke *et al.*, 2014 ▸), the mechanical ruling of blazed gratings is very challenging and has not yet been substituted by a more simple method. An alternative approach was recently tested at State University of Illinois to mechanically rule gratings with an atomic force microscope (AFM) (Abbamonte & MacLaren, 2014 ▸), but even this method requires a dedicated and complex ultra-precise etching technique and the achievable grating sizes seem to be limited.

Blazed gratings manufactured on the basis of wet anisotropic etching of asymmetrically oriented crystals (Fujii *et al.*, 1980 ▸; Voronov *et al.*, 2010 ▸) have shown promising results for small dimensions of approximately 30 mm × 30 mm and at a high groove density above 5000 lines mm^−1^ (Voronov *et al.*, 2014 ▸). Grating sizes up to 120 mm × 30 mm using this method seem to be realistic (Voronov *et al.*, 2017 ▸). Our own experience with this method has shown limitations on the availability and cost of single-crystal silicon of dedicated orientation in particular for very small blaze angles of 0.2° to 0.5° as well as on the required substrate finishing quality (sub-surface damage) and substrate geometry.

This paper describes the technological processes necessary for a successful manufacture and metrology of gratings starting with the preparation and metrology of grating blanks (§2), and the required nano-technology for laminar gratings (§3) and blazed gratings (§4). The transfer of the grating structure into the substrate by ion etching is described in §5, and the final performance test of the finished gratings by at-wavelength metrology (reflectometry) and comparison with simulated efficiency data are treated in §6.

## 2. *Ex situ* characterization of grating blanks and gratings   

**Figure 1:**
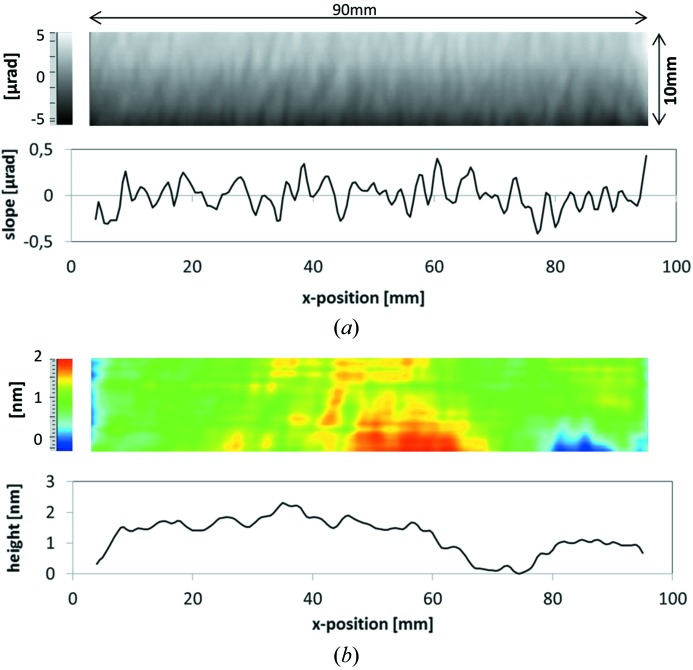
(*a*) Topography of a state-of-the-art grating blank in terms of 2D meridional slope (absolute data) and profile of the slope at the centre line. (*b*) Corresponding 2D height data and height profile at the centre line.

**Figure 2:**
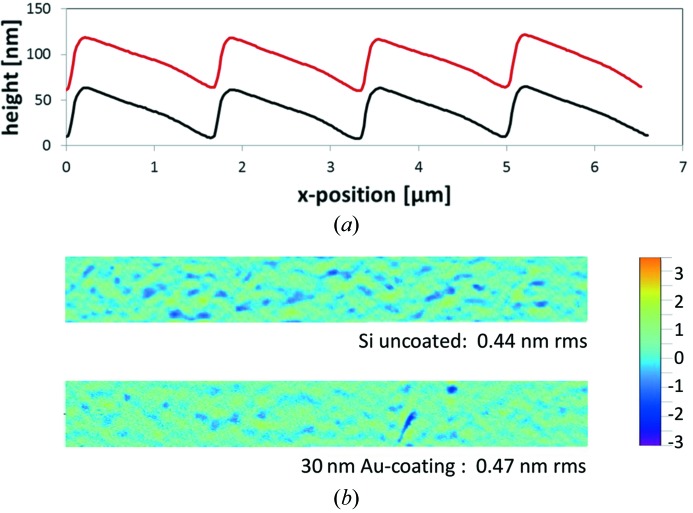
Comparison of the groove state by means of AFM measurements on a 600 lines mm^−1^ grating with 2° blaze angle. (*a*) Groove profile before (red curve) and after coating with 30 nm Au (black curve). (*b*) Micro-roughness before and after coating as measured on one groove section for a field of view of 8 µm × 1 µm. These measurements were performed with a Bruker-SIS-Ultraobjective AFM.

A precise knowledge of the initial quality of a substrate before manufacturing a grating is a basic requirement. By dedicated instrumentation the substrate needs to be characterized within the entire range of spatial frequencies: (i) slope errors corresponding to long-spatial frequency error (aperture length > 1 mm^−1^), (ii) mid-spatial frequency error (1 mm^−1^ to 1 µm^−1^) and (iii) high-spatial frequency error (1 µm^−1^ to 10 nm^−1^) which is often described as micro-roughness (VDI/VDE 5575, 2011*a* ▸). While the substrate slope error is determined by direct slope measuring deflectometry (Siewert *et al.*, 2014 ▸, 2016 ▸), micro-roughness is inspected with a white-light interferometer (Wyant, 2002 ▸) and/or an AFM (Binnig *et al.*, 1986 ▸). These inspections allow the state of the surface figure and finish to be characterized as well as the level of contamination of the substrate. Small particles (<100 nm diameter) or a contamination layer remaining after the substrate manufacturing can be revealed by an AFM measurement. During manufacturing, additional AFM measurements are required to check the intermediate state after each process step. This includes a check of the ruling coating (Au in our case) before and after the ruling process as well as after the IBE and of the final coated grating. As an illustration, Fig. 1(*a*) ▸ shows a two-dimensional slope map and the center line in the meridional direction of a state-of-the-art blank for a plane grating; it has a residual slope error of 150 nrad r.m.s., a micro-roughness of <0.2 nm r.m.s. and a meridional radius of >500 km as measured by slope mapping with the BESSY-NOM (Siewert *et al.*, 2005 ▸, 2011 ▸). The surface topography is obtained by integration of the slope data (Fig. 1*b* ▸). Fig. 2 ▸ shows the results of AFM measurements on a blazed grating of 600 lines mm^−1^ with blaze angle 2° obtained after the IBE process with the final blaze and with the final Au coating of thickness 30 nm. It is shown that the final coating of a grating with a single-layer material does not cause a change of the groove profile or of the micro-roughness on the grooves. The groove placement accuracy control for constant pitch or for line density variation (in the case of a VLS grating) is measured by the use of a groove density measurement set-up (VDI/VDE 5575, 2011*b* ▸) available at HZB. A fast qualitative check to monitor this parameter is possible by aligning the grating under the Littrow condition in front of a Fizeau phase-shifting interferometer which allows viewing of the quality of the wavefront in higher diffraction order (Loechel *et al.*, 2013 ▸).

## 3. Laser interference lithography for laminar gratings   

Laminar gratings can be manufactured using a lithographic process to fabricate an etching mask and a subsequent transfer of the grating structure into the silicon substrate by IBE (Wolferen & Abelmann, 2011 ▸). To ensure the required quality during the exposure, the environmental control on temperature, humidity as well as dust particles to achieve a high setup stability are most important issues. The humidity must stay below 40% and be stable to within ±5% during the exposure time. The thermal stability of the clean-room laboratory is specified to ±100 mK day^−1^ while the particle concentration is ISO Class 4.

In a first step a resist layer (200 nm) is deposited on the surface of the substrate by spin-coating and then baked at a temperature of 100°C in an oven at atmospheric pressure (Lemke *et al.*, 2014 ▸). The etching mask is made by laser interference lithography. To obtain constant line-spacing gratings, plane-wave setups are realised. The exposure wavelength λ and the incidence angle θ with respect to the grating normal determine the achieved grating period *p*,
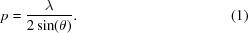
In our case the laser wavelength and the maximum incidence angle of θ ≤ 65° limit the maximum possible line density to 3960 lines mm^−1^ or 4100 lines mm^−1^ for a solid-state laser (DPSS, 300 mW, 457 nm, Acal BFi Germany GmbH) or a HeCd gas laser (442 nm, 180 mW, Kimmon), respectively, used as a source for the exposure process (Lemke *et al.*, 2014 ▸). In the case of a deep UV DPSS CW laser (266 nm), a groove density up to 6800 lines mm^−1^ could be reached. After exposure and development the resist layer is etched away with an oxygen plasma to remove the rest of the resist. During this etching process the groove width is usually increased, so that the groove width to spacing ratio is changed. This ratio is important for the performance of the grating and is normally defined in the grating specification.

A challenge of the patterning process for XUV gratings is the demanding requirements on the precision of the grating parameters, such as the tolerances in the constancy of the line density especially if a VLS grating is requested. The groove width to spacing ratio must be matched during the exposure step, while the depth control is important during the pattern transfer in the subsequent etching step. Large patterning areas and substrate sizes as well as dedicated substrate shapes are additional factors, which are mostly limited by the capability of the processing equipment.

Different optical setups using planar or spherical waves were used to generate interference patterns and to expose the resist layer. Exposure times between several minutes and about two hours were used. During exposure the refractive index of the air in the beamline must be kept constant. This means that air agitation, noise, change in temperature, air pressure or humidity influence the quality of the generated pattern strongly.

**Figure 3:**
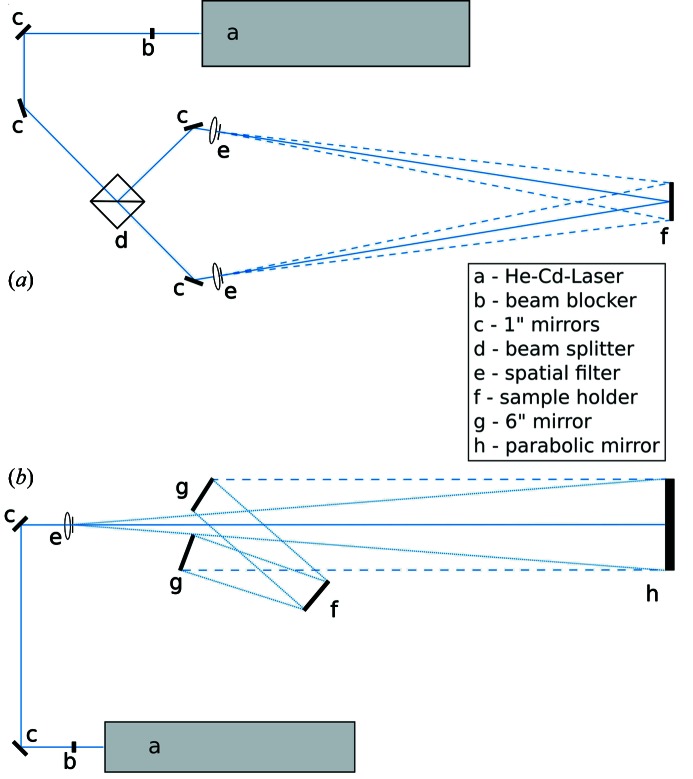
(*a*) Schematic diagram of a spherical-mirror-based setup for holographic exposure. (*b*) Schematic diagram of a setup using a parabolic mirror.

**Figure 4:**
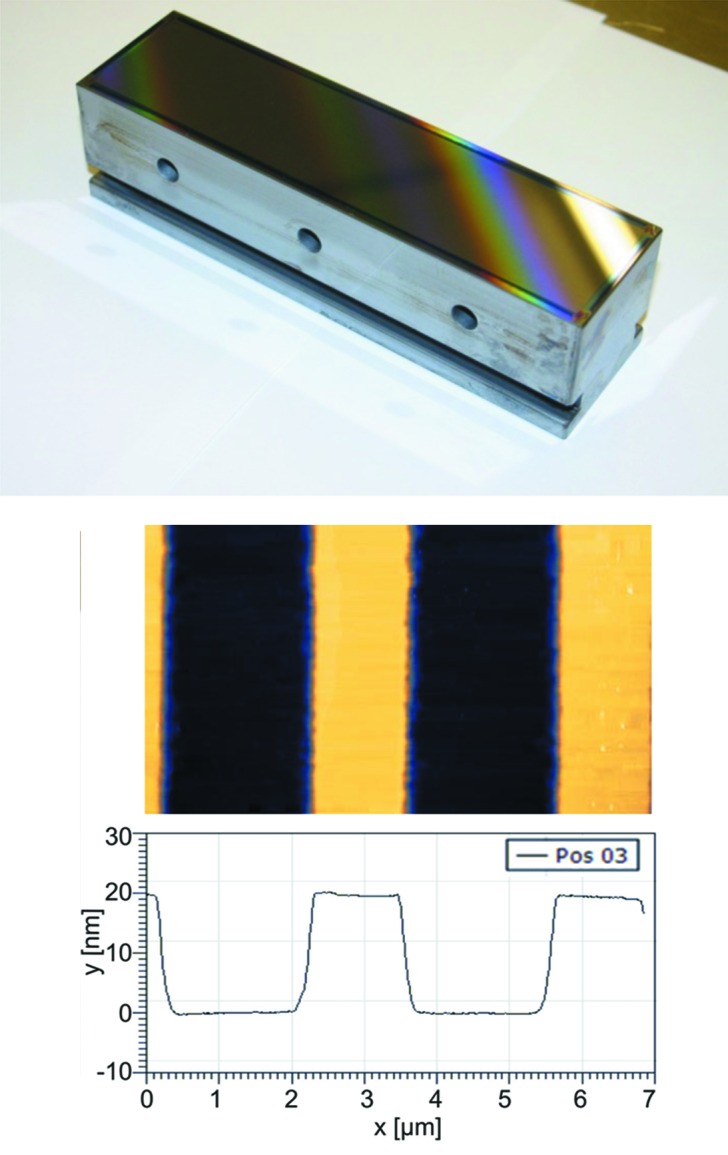
Top: laminar grating (300 lines mm^−1^ groove density) for the I08 SXM beamline at Diamond; patterned resist on a Si substrate of length 150 mm. Bottom: AFM profile measurement at the laminar grating; footprint and AFM profile showing 20 nm groove depth.

A schematic optical layout of the laser interference lithography setup is shown in Figs. 3(*a*) and 3(*b*) ▸ for a spherical and parabolic setup, respectively. First test exposures with the spherical wave setup, Fig. 3(*a*) ▸, were used for samples on silicon wafers. The setup using the parabolic mirror, Fig. 3(*b*) ▸, was applied for the grating shown in Fig. 4 ▸ and discussed in Fig. 15. Under optimum environmental conditions, large groove densities of more than 4000 lines mm^−1^ could be generated. Both setups are fundamental for the fabrication of laminar gratings. If no high constancy in the groove density is required, the setup using spherical waves delivers high-quality gratings. Substrates up to 6-inch in diameter were successfully exposed with such a setup. For higher demands in the constancy of the groove density, such as for gratings for synchrotron applications, a setup with a parabolic mirror (Fig. 3*b* ▸) delivers better results. Such a setup, however, requires high-precision mirrors to avoid stray light and speckle effects which may cause an overlay of a second interference pattern and thus local exposure density variations which result in local groove width and depth variations. To finalize such a grating after plasma etching the grating pattern is transferred to the silicon substrate by IBE, which is described in §5. Finally, the grating will be coated with a dedicated single layer or a multilayer to provide the required performance. Fig. 4 ▸ shows a photograph of a laminar grating of 300 lines mm^−1^ groove density with patterned resist after exposure and an AFM image of the final profile after ion beam treatment.

## 4. On the mechanical ruling of gratings   

**Figure 5:**
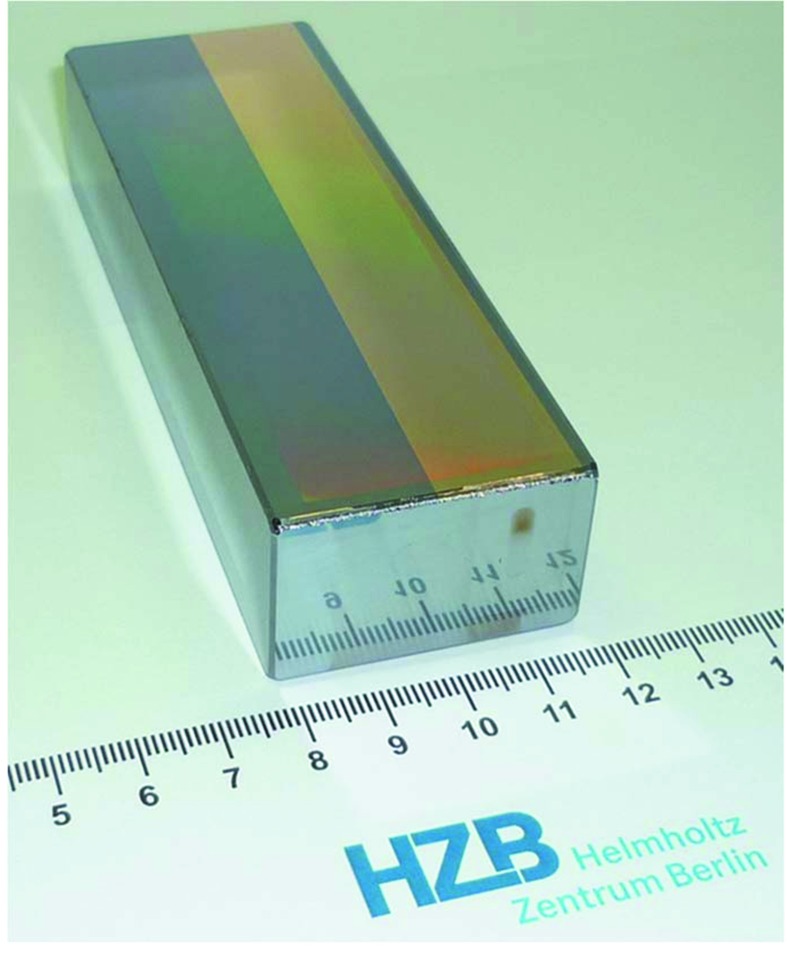
First synchrotron grating ruled at HZB (600 lines mm^−1^, blaze angle 2°) for the SX700 monochromator PM-1 of the new Optics beamline at BESSY-II. In this state the grating substrate has one half of the aperture uncoated (left) and one half coated with 30 nm Au (right).

**Figure 6:**
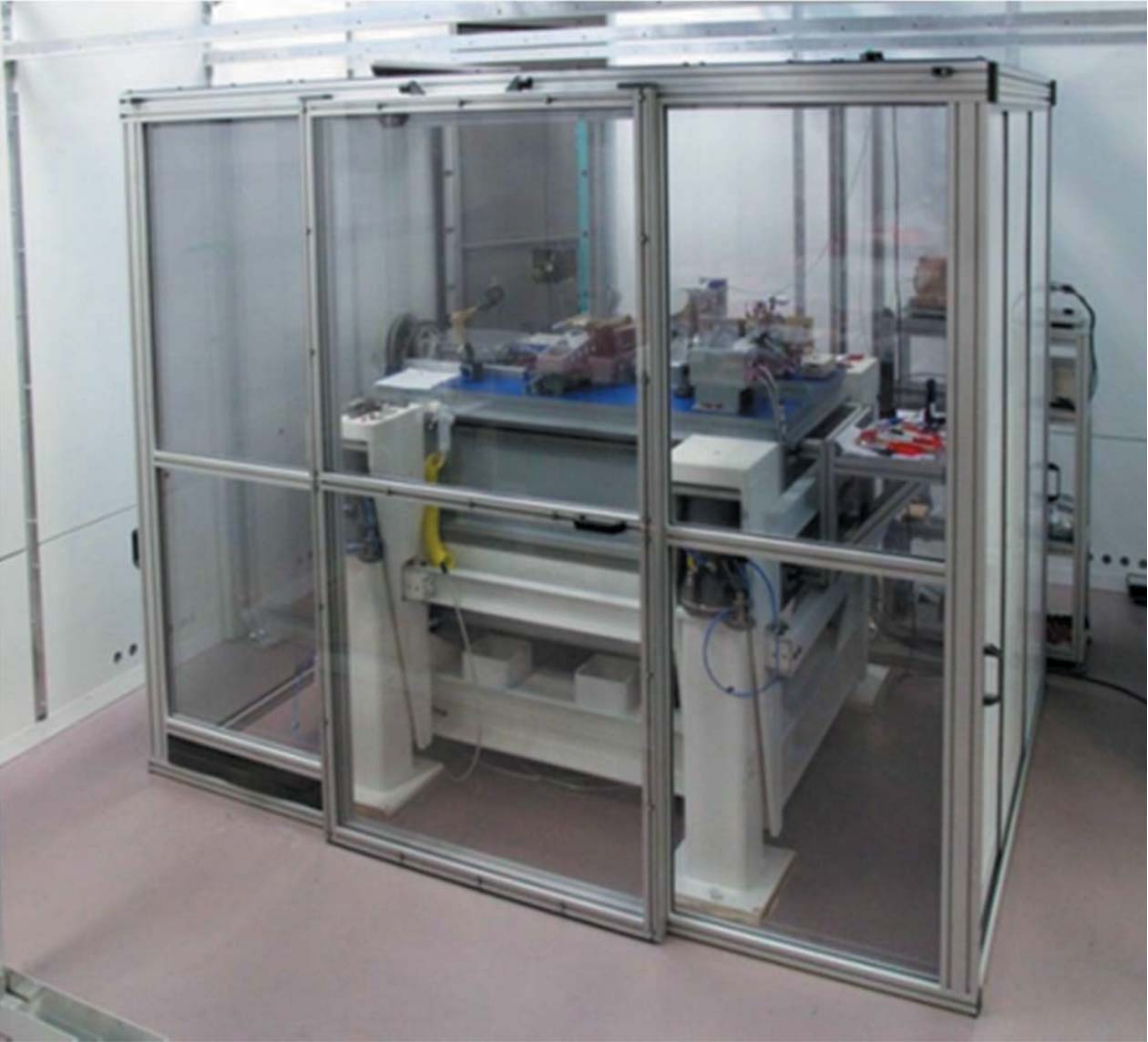
Ruling machine GTM-6 on a passive vibration damping support and covered by a double-wallet inner hutch (walls of the environmental controlled outer hutch are seen in the background).

The principle of mechanical ruling for manufacturing blazed gratings has been known for a long time (Fraunhofer, 1823 ▸; Roland, 1882 ▸; Michelson, 1915 ▸). The technique has been improved over the years and was successfully applied, for example, by Carl Zeiss Optronics GmbH in Oberkochen until 2010. After the decision of the company to close down the production of blazed gratings, the established Zeiss technology line was transferred to the HZB in Berlin and was upgraded considerably. In 2013, one of the first successfully blazed gratings (see Fig. 5 ▸) was ruled with the Zeiss GTM-6^1^ ruling machine (see Fig. 6 ▸). It has 600 lines mm^−1^ and 2.0° blaze angle and is now installed in the Optics beamline at BESSY-II (Sokolov *et al.*, 2016 ▸).

The ruling of gratings is carried out by use of diamond tools. Diamond is still the only known material that can provide a sufficient long-term wear-resistance to rule grooves up to a total groove length of several kilometres, which is required for real-size gratings (typically 30 mm × 100 mm) and groove densities up to 3000 lines mm^−1^.

**Figure 7:**
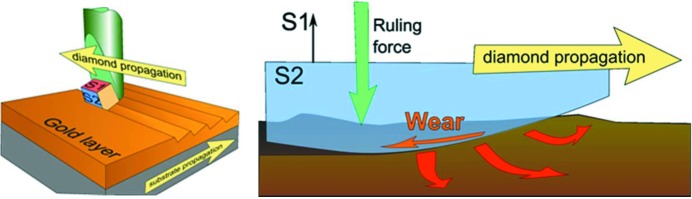
Schematic diagram of the ruling process with a diamond tool for a blazed grating. The substrate and diamond propagation directions are indicated by arrows. S1 and S2 denote the crystal orientation of the diamond. The right-hand figure is an enlarged view into the process of groove manufacturing in the Au layer indicating the wear forces and directions.

Fig. 7 ▸ shows a schematic of the ruling process. To rule a groove pattern into the Au-coated grating surface, two orthogonal motions are required. (i) The grating blank translates continuously with very low velocity along its length, perpendicularly to the groove direction. (ii) A diamond tool translates perpendicular to this motion on the grating blank along the groove direction. After finishing one groove at the grating end the diamond tool is lifted up and moved back to the starting point at the opposite side of the grating. Then the diamond tool is pushed down again and starts to shape the next groove. To achieve the desired groove profile the diamond needs to be precisely oriented, aligned and force-controlled for the specified groove profile and depth. That is why ruling can be performed in one direction only.

The movement of both grating blank and ruling diamond needs to be controlled within a few nanometres to guarantee equally spaced grooves with high parallelism along the total grating length. Depending on the specified line density the velocity of the grating blank varies between a few nm s^−1^ and 10 mm s^−1^. Defined by these parameters, the total ruling time of a grating can take up to several days or even several weeks. To provide the required accuracy for the positioning and movement of the substrate carriage and ruling tool carriage of the GTM-6, a laser interferometric feedback control is applied as proposed by Harada in earlier work (Harada *et al.*, 1974 ▸).

**Figure 8:**
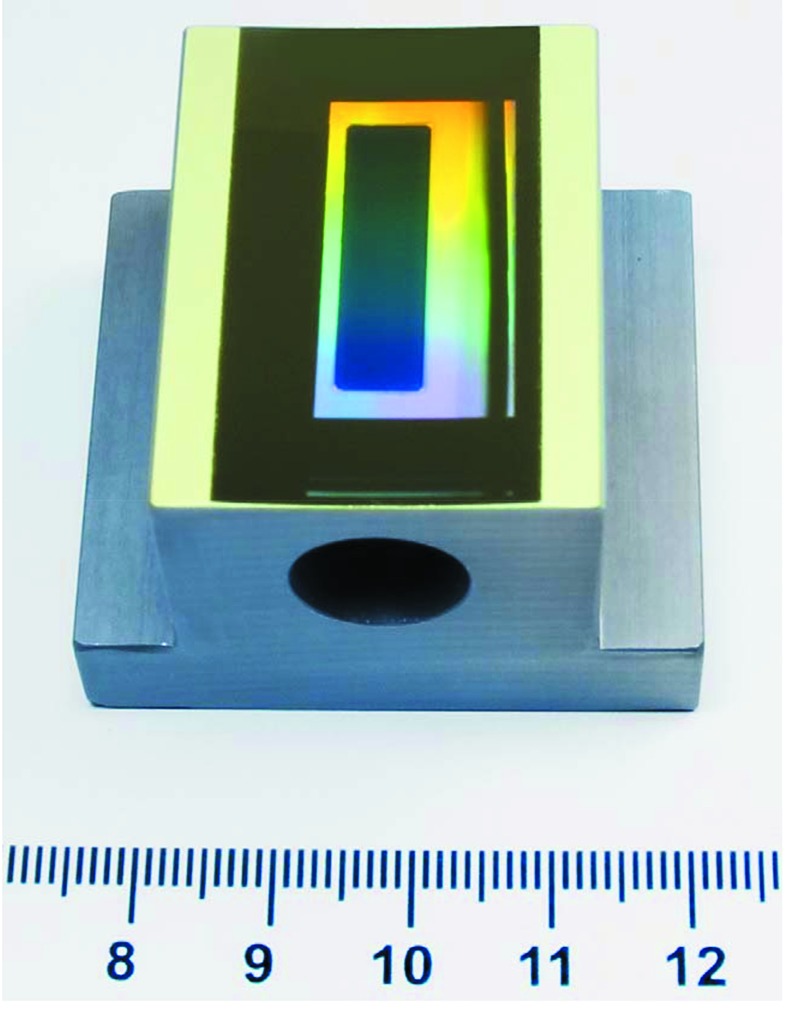
Toroidal VLS grating for soft X-ray self-seeding at LCLS (Stanford). Median line density 1123 lines mm^−1^, blaze angle 1.4°, surface roughness 0.4 nm r.m.s. (with parameters of the toroidal substrate: meridional radius *R* = 195 m, sagittal radius *r* = 0.18 m.

The ruling of a grating is performed on a surface that needs to be soft enough to accept a local deformation. Thus the grating substrates are coated with a ductile material. Known materials are speculum metal (an alloy of tin and copper), vacuum-deposited aluminium or gold (Loewen, 1997 ▸; Kröplin, 2000 ▸). We have decided to apply Au coatings as established with great success by the Zeiss company. Ruling into gold has the advantage that no additional lubricant is needed (*e.g.* silicon oil is required for ruling into aluminium). Large blaze angles of 4–10° can be realised directly by the ruling process. Smaller blaze angles require a further process step like IBE. This is explained in the following section. Mechanical ruling allows gratings to be fabricated on plane as well as on slightly curved substrates of spherical or toroidal shape. Fig. 8 ▸ shows a toroidal grating made for the soft X-ray self-seeding process of the FEL at the Linac Coherent Light Source (LCLS) in Stanford (Cocco *et al.*, 2013 ▸; Ratner *et al.*, 2015 ▸). The hole in the substrate bulk allows the electron bunches of the FEL to pass through to interact with photons of defined energy behind the grating.

To achieve high-performance gratings the ruling needs to be performed under defined environmental conditions regarding thermal stability and vibration. Already in the 1950s a thermal stability of 10–20 mK day^−1^ was reported (Kröplin, 2000 ▸), a condition which meets our present-day laboratory environment as well. In addition, our ruling machines are placed on vibration damping mechanics; see Fig. 6 ▸, which shows the GTM-6 on its passive vibration damping frame (white coloured) covered with a double-walled inner hutch.

At the ruling machine GTM-6 the maximum grating length is limited to 170 mm. Upcoming FEL facilities like the European XFEL require plane gratings of significantly larger dimension of up to 500 mm × 30 mm (Vannoni *et al.*, 2013 ▸). These gratings are designed to have low groove densities between 50 lines mm^−1^ and 150 lines mm^−1^ with very small blaze angles of less than 0.3°. Thus a new ruling machine has been specified, ordered and delivered. The new ruling machine, GTM-24, allows gratings to be ruled up to a length of 24 inch (600 mm) and a maximum width of 300 mm. The smallest blaze angle to be realised by means of mechanical ruling at the GTM-24 will be about 3°. Then the final blaze angle is realised by IBE (see §5).

Note that to rule a grating with the largest GTM-24 design parameters in length (600 mm), width (300 mm) and groove density (6000 lines mm^−1^) would take several years! Thus, such gratings will remain unrealistic for manufacturing even with the new GTM-24. Gratings as specified for the European XFEL are estimated to be ruled within one to three weeks, which is a processing time similar to the GTM-6, which can be seen as realistic. Note that the maximum ruling time realised at the GTM-6 is 25 days so far.

**Figure 9:**
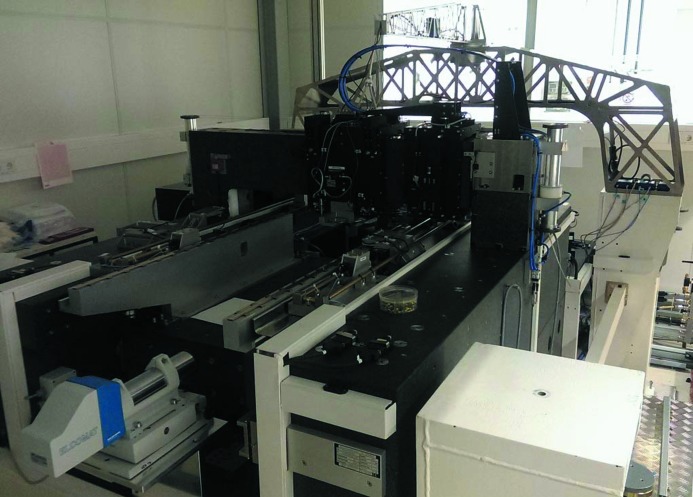
The grating ruling machine GTM-24 during commissioning at HZB.

Fig. 9 ▸ shows the delivered GTM-24 ruling machine during commissioning at HZB. The design of the GTM-24 follows the concept of the GTM-6. It carries the grating substrate face-up on a continuously slowly moving carriage and the diamond ruling tool is placed downwards on a second carriage to shape the grooves perpendicular to the substrate propagation (see Fig. 7 ▸). For *in situ* diagnostics of the alignment of the ruling tool an AFM is located at the ruling carriage close to the ruling tool. It can be easily moved to the ruling area to measure the groove profiles. The GTM-24 has a total weight of 14 tons and thus has a very low eigenfrequency as well as a sluggish response to thermal instabilities. The machine is embedded in a passive damping frame on a separated base and is covered by two hutches: a passive double-walled inner hutch and a climate-controlled outer hutch. Currently the control software has been developed and in the medium term the commissioning of the new ruling machine is intended. In a second step a software upgrade will allow the ruling of VLS gratings.

## 5. The final groove profile: IBE of the grating   

**Figure 10:**
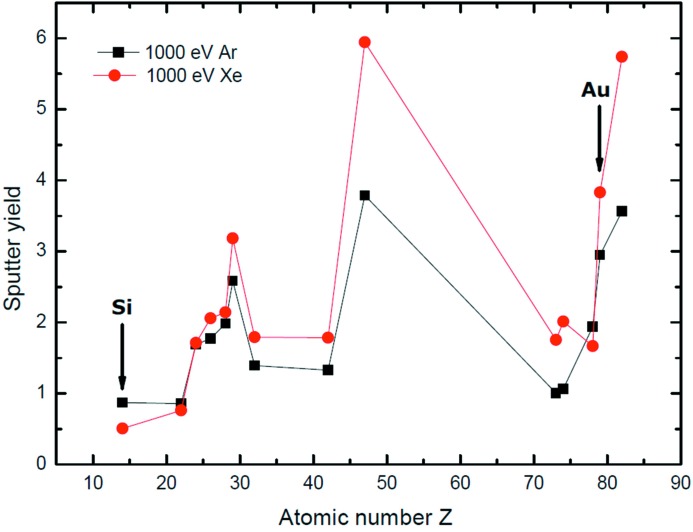
Ion beam etching. Calculated sputter rates of selected elements for sputter ions Ar^+^ and Xe^+^ at 1 keV sputter energy and 90° incidence angle of the ion beam to the surface. Si and Au are indicated by arrows.

**Figure 11:**
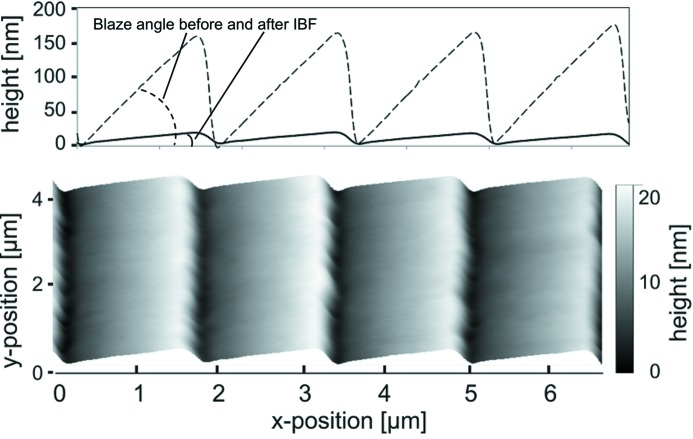
AFM topography measurement on a 600 lines mm^−1^ blazed grating manufactured with the GTM-6. Top: surface profiles of the grating before (dashed line) and after (solid line) ion beam treatment. Bottom: 3D visualization of the corresponding AFM measurement after IBE.

State-of-the-art gratings of laminar as well as of blazed groove profile are etched into the grating substrate of single-crystal silicon by use of IBE (Albert *et al.*, 1994 ▸; Nelles *et al.*, 2001 ▸). In a first approach we investigated the etching process for blazed gratings with a Kaufman source at the Leibniz Institut für Oberflächenmodifizierung (IOM) in Leipzig. Different etching gases like Ar^+^, Xe^+^, N^+^ and O^−^ were investigated and all of them showed usability for the desired process. Each gas provides different etch rates, based on the individual selectivity for Au, Si and for the binding layer material Cr. Fig. 10 ▸ shows the calculated sputtering yields (etch rates) of Au and Si using Ar^+^ and Xe^+^ ions for normal ion incidence (Seah, 2005 ▸; Seah *et al.*, 2005 ▸). By combining different gases, a wider parameter space for this manufacturing step can be achieved. A further process parameter is the etching angle between ion beam source and substrate. This angle needs to be optimized for each individual blazed grating to achieve the specified blaze angle as well as to avoid, for example, trenching effects. The transfer of the grating structure from the coating to the substrate material with subsequent coating does provide several advantages. It has a higher mechanical robustness, and in general the coating can be removed by dedicated chemistry without destroying the grating pattern and it can be recoated again. In particular, it is the only way to achieve shallow blaze angles of less than approximately 3°. Fig. 11 ▸ shows the state of the AFM topography of a blazed grating with 600 lines mm^−1^ before and after IBE. The blaze angle was reduced by a factor of ten from an initial 6.6° blaze angle to a final blaze angle of 0.62°. The micro-roughness was considerably reduced from 0.56 nm r.m.s. to 0.12 nm r.m.s.

**Figure 12:**
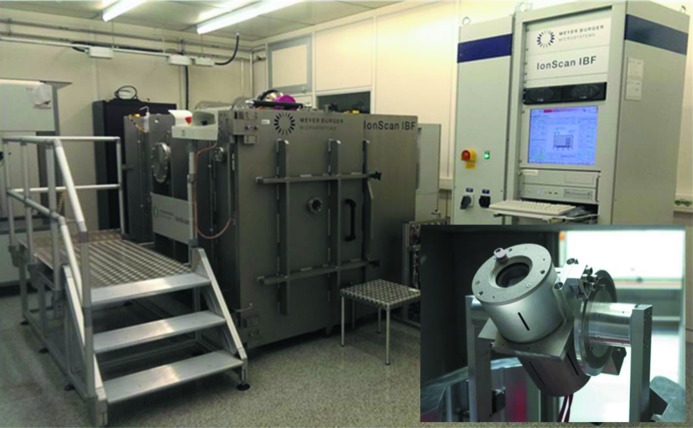
Ion beam figuring system at HZB equipped with a 2D-scanning HF source. The insert in the bottom right-hand corner shows the HF source in a tilted position. The total tilt-range is ±30°.

**Figure 13:**
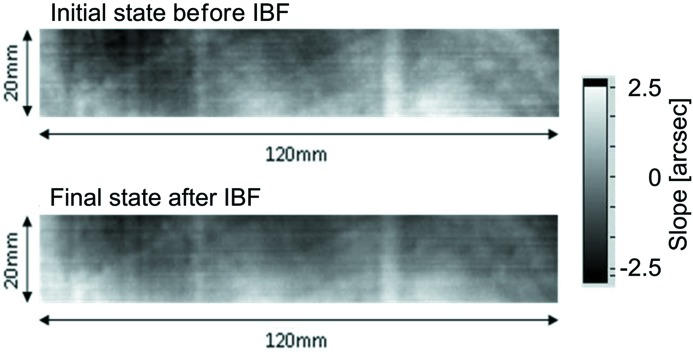
Substrate topography of a Si substrate measured with the BESSY-NOM before (top) and after (bottom) ion beam treatment with Ar^+^ ions at a 90° sputter angle. The surface topography including the higher-spatial-frequency surface pattern remains unchanged.

At HZB, currently two ion beam figuring (IBF) systems are available. The Zeiss technology line includes a system with a tiltable and rotatable sample holder and a fixed Kaufman source (Kaufman, 1974 ▸, 1978 ▸) of 270 mm diameter which has 95% homogeneity of the etching rate over a length of 230 mm. For larger grating sizes (up to 500 mm × 100 mm) a new IBF system with a two-dimensional-scanning tiltable radiofrequency (13.54 MHz) excited ion beam source (45 mm diameter) was developed. The sample to be etched is mounted face-down inside the chamber. Fig. 12 ▸ shows this new IBF system in our cleanroom environment. Fig. 13 ▸ shows a test result for the homogeneity of the etching process (Ar^+^ ions) applied to a Si substrate of 120 mm × 20 mm size. The slope error was measured with the BESSY-NOM before and after ion beam treatment. The global substrate topography as well as the residuals in the spatial frequency range of a few mm^−1^ were well preserved during the etching process which is an important criterion for the suitability of this method.

## 6. At-wavelength metrology: final characterization of gratings   

**Figure 14:**
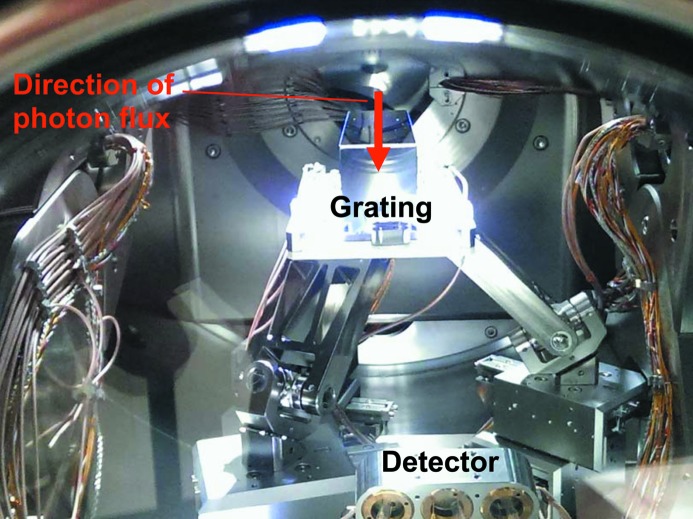
At-wavelength-metrology. A grating within the UHV chamber of the reflectometer mounted on top of the tripod alignment stage. Some photodiode detectors are seen at the front and part of the cabling assembly of the azimuthal rotation stage at the sides.

**Figure 15:**
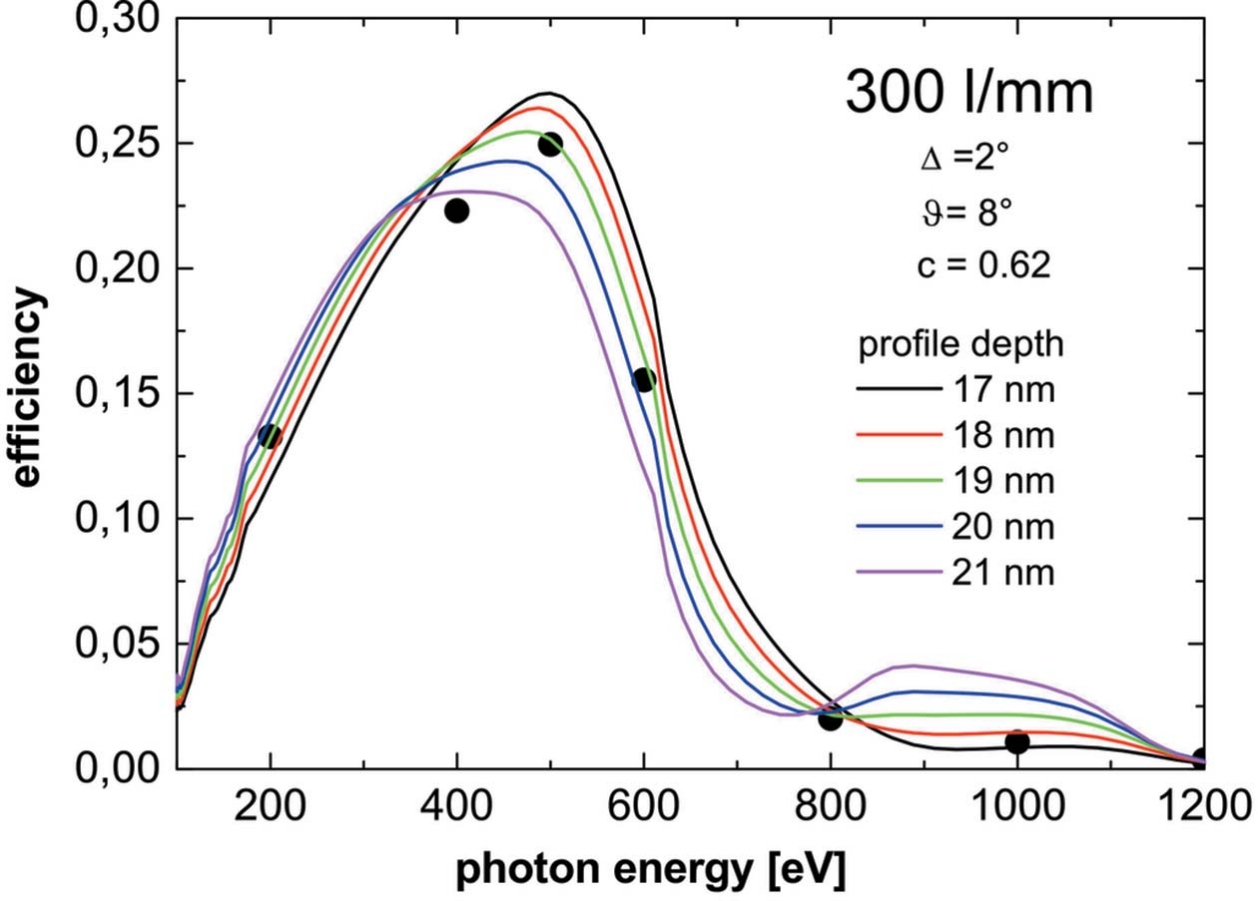
Measured efficiency data for a 300 lines mm^−1^ laminar Si-grating with an aspect ratio of 0.62 and a trapezoidal angle of 8° obtained at 2° incidence angle. Simulated curves were obtained with the *REFLEC* code with the profile depth as parameter. These results confirmed the profile depth measured by AFM.

**Figure 16:**
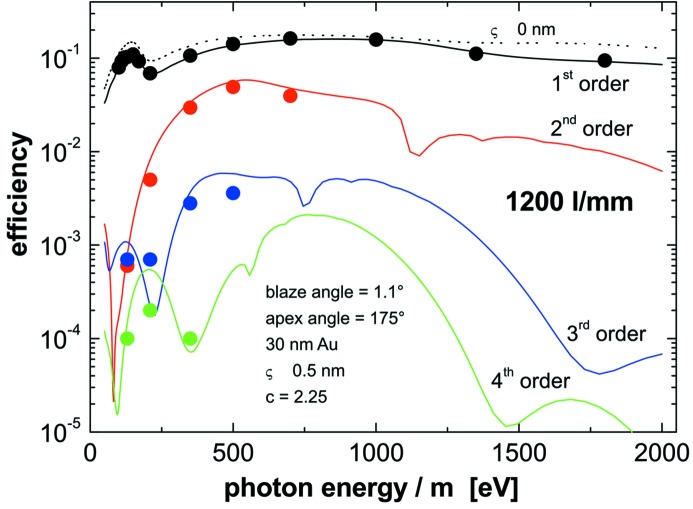
Efficiency of a 1200 lines mm^−1^ blazed grating operated at *c* = 2.25 in the *m* = first to fourth diffraction order as function of the *m*th-order energy in comparison with simulation (including roughness) with the *REFLEC* code. The grating has a blaze angle of 1.1° and is coated with 30 nm Au (without binding layer).

**Figure 17:**
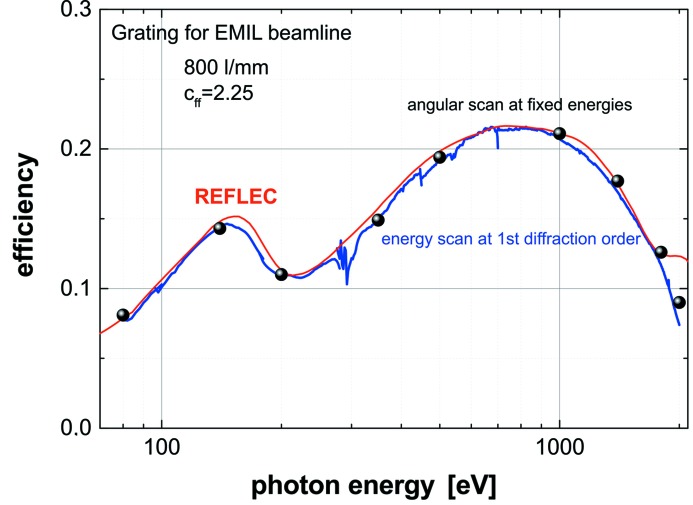
Diffraction efficiency of an 800 lines mm^−1^ blazed grating manufactured for the EMIL beamline project at BESSY-II measured in the first diffraction order. Points: integrated diffraction efficiency derived from angular scans; blue curve: on-the-fly energy scan with simultaneous scan of the diffraction angle and detector angle according to the grating equation; red curve: simulation with the *REFLEC* code.

At-wavelength metrology is a powerful and indispensable tool for the characterization and final control of soft X-ray optical elements. Since the optical constants of the coating materials involved depend on wavelength, information on reflectivity or diffraction efficiency at a certain energy can be obtained only by this method and cannot be deduced from any other diagnostics results. The at-wavelength inspection of a grating is the final test drive before its installation in a monochromator or spectrometer. To provide such information we have set up a new Optics beamline (Sokolov *et al.*, 2016 ▸; Schäfers *et al.*, 2016 ▸) and a new versatile UHV reflectometer (Eggenstein *et al.*, 2014 ▸) as a permanent endstation at the BESSY-II facility. The collimated Plane Grating Monochromator beamline (c-PGM) is located on a bending magnet and allows characterization of gratings in the energy range from 20 eV to 2000 eV in s- and p-polarization geometry and with high flexibility in beamline operation modes (high flux, high resolution, high order suppression mode). The SX700 monochromator of the beamline is equipped with two blazed gratings of our own production with respective line densities of 600 and 1200 lines mm^−1^. Higher beamline diffraction orders can be eliminated very efficiently (<0.05%), (i) by variation of the c-factor of the SX700, (ii) by use of adequate high-order filters in the beamline (Schäfers *et al.*, 2016 ▸) and (iii) by a recently commissioned higher-order suppressor (Sokolov *et al.*, 2018 ▸). Fig. 14 ▸ shows a grating under test mounted on top of the tripod alignment stage in the UHV reflectometer. As an illustration, Fig. 15 ▸ gives the first-order integrated efficiency data measured for a laminar grating of 300 lines mm^−1^ in comparison with calculation using the *REFLEC* code (Schäfers & Krumrey, 1996 ▸). The calculated curves confirmed the profile depth of 19 nm measured with an AFM. Fig. 16 ▸ shows the integrated efficiency for a 1200 lines mm^−1^ blazed grating set at a c-factor of 2.25 for the first to fourth diffraction order as a function of energy, again in excellent agreement with simulated data using the *REFLEC* code. The high quality of the gratings produced in-house with the GTM-6 is also demonstrated in Fig. 17 ▸. Here the first-order integrated efficiency is displayed as a function of energy. For a quick survey the diffraction angle on the grating and the detector angle were continuously scanned together with the photon energy of the primary beam according to the grating equation (blue curve). The results are in close agreement with angular scans at fixed energy (points) and with simulation (red curve).

## 7. Conclusions and outlook   

A technology line for the production and characterization of blazed and laminar gratings has been established at Helmholtz Zentrum Berlin. The existing equipment allows gratings of constant as well as of variable line spacing to be manufactured for application in the IR, UV, VUV and soft X-ray range which is of utmost importance to the synchrotron radiation and FEL communities. We assume that further fields of research such as X-ray astronomy and laser science will benefit from this work as well. At-wavelength performance tests of different types of gratings at the Optics beamline have shown excellent efficiency values close to the simulated ones. The versatile reflectometer at the Optics beamline is available as a user facility. Beam time proposals are welcome.

Future work will concentrate on the commissioning of the new ruling machine GTM-24 which is still in progress. The GTM-24 will allow ruling of large gratings up to 500 mm in length as required, for example, for the European XFEL beamlines. A further topic of future development is to cover the ‘difficult spectral region’ between 1 and 5 keV photon energy with gratings which have a dedicated multilayer coating (Senf *et al.*, 2016 ▸).

## Footnotes

1 GTM stands for Gitter-Teil-Maschine (grating ruling machine). The number denotes the maximum grating dimension in inches.
